# Genetic causal role of body mass index in multiple neurological diseases

**DOI:** 10.1038/s41598-024-57260-2

**Published:** 2024-03-27

**Authors:** Xie Wang, Hong Chen, Ze Chang, Juan Zhang, Daojun Xie

**Affiliations:** 1 Anhui University of Chinese Medicine, Hefei, Anhui 230038 China; 2 Xiyuan Hospital, China Academy of Chinese Medical Sciences, Beijing, 100089 China; 3 Department of Neurology, The First Affiliated Hospital of Anhui University of Traditional Chinese Medicine, 117 Meishan Road, Hefei, Anhui 230031 China

**Keywords:** BMI, Neurological diseases, Mendelian randomization analysis, MS, IS, Genetics, Neurology

## Abstract

Body mass index (BMI) is a crucial health indicator for obesity. With the progression of socio-economic status and alterations in lifestyle, an increasing number of global populations are at risk of obesity. Given the complexity and severity of neurological diseases, early identification of risk factors is vital for the diagnosis and prognosis of such diseases. In this study, we employed Mendelian randomization (MR) analysis utilizing the most comprehensive genome-wide association study (GWAS) data to date. We selected single nucleotide polymorphisms (SNPs) that are unaffected by confounding factors and reverse causality as instrumental variables. These variables were used to evaluate the genetic and causal relationships between Body Mass Index (BMI) and various neurological diseases, including Parkinson’s Disease (PD), Alzheimer's Disease (AD), Amyotrophic Lateral Sclerosis (ALS), Multiple Sclerosis (MS), Ischemic Stroke (IS), and Epilepsy (EP). The Inverse Variance Weighted (IVW) analysis indicated that there was no significant causal relationship between Body Mass Index (BMI) indicators and PD (*P*-value = 0.511), AD (*P*-value = 0.076), ALS (*P*-value = 0.641), EP (*P*-value = 0.380). However, a causal relationship was found between BMI indicators and MS (*P*-value = 0.035), and IS (*P*-value = 0.000), with the BMI index positively correlated with the risk of both diseases. The Cochran’s Q test for MR-IVW showed no heterogeneity in the MR analysis results between the BMI index and the neurological diseases (*P* > 0.05). The Egger intercept test for pleiotropy revealed no horizontal pleiotropy detected in any of the neurological diseases studied (*P* > 0.05). It was found that there was no causal relationship between BMI and PD, AD, ALS, EP, and a genetic causal association with MS, and IS. Meanwhile, the increase in BMI can lead to a higher risk of MS and IS, which reveals the critical role of obesity as a risk factor for specific neurological diseases in the pathogenesis of the diseases.

## Introduction

Body Mass Index (BMI), a prevalent metric for assessing obesity, has been extensively employed for its simplicity, convenience, and high sensitivity in detecting fat accumulation in clinical settings^1,2^. With the ongoing evolution of economic standards and shifts in dietary habits and lifestyles, a significant number of children and adults are confronted with the risk of obesity^3^. An increasing body of research has investigated the health implications of elevated BMI, revealing that a high BMI not only elevates the risk of various diseases but also augments mortality rates^4–6^. Numerous studies have corroborated the association between a high BMI and an increased likelihood of developing specific conditions, such as type 2 diabetes, hypertension, and coronary heart disease^7,8^. Moreover, a high BMI contributes to the onset of musculoskeletal disorders, encompassing rheumatoid arthritis and psoriatic arthritis, as well as neoplastic growths^9,10^.

Neurological diseases encompass a wide array of lesions that affect the human nervous system. These include but are not limited to, cerebrovascular disorders, neurodegenerative conditions, demyelinating lesions, infectious neurological diseases, oncological disorders, metabolic neurological diseases, and hereditary neurological illnesses. As society ages, neurological diseases have emerged as a leading cause of mortality and disability among humans. Among them, Parkinson’s Disease (PD) and Alzheimer’s Disease (AD) are the two most common neurodegenerative diseases. The etiology of Parkinson’s disease is not yet clear and is believed to be related to age, gender, smoking, alcohol consumption, vitamin D exposure, pesticide use, uric acid levels, and cranial brain injuries^11–15^. Its main pathological basis is the concentration of striatal dopamine and the presence of neuronal Lewy bodies^12,13^. Alzheimer’s disease is considered to be related to age, infection, and environmental factors^16,17^, and its main pathology includes the deposition of β-amyloid protein, phosphorylation of tau protein, inflammatory mechanisms, and mitochondrial dysfunction^18^. Amyotrophic Lateral Sclerosis (ALS) is a neurodegenerative disease affecting both upper and lower motor neurons. The etiology and pathogenesis of ALS remain largely unclear. Pathological studies have reported the aggregation and accumulation of ubiquitinated protein inclusion bodies within motor neurons^19^. Multiple Sclerosis (MS), is an immune-mediated demyelinating disease of the central nervous system, distinguished by its spatial and temporal multiplicity. To date, the etiology of MS remains elusive, though it is postulated to be linked to immune responses triggered by infections, including those caused by Epstein–Barr Virus (EBV), human herpesvirus type 6, and measles virus, among others^20^. Ischemic stroke (IS), a prevalent cerebrovascular condition, has seen an increasing trend in recent years. It is widely believed to be closely associated with factors such as advancing age, hypertension, hyperlipidemia, diabetes mellitus, smoking, alcohol consumption, cardiac diseases, and vascular inflammatory conditions^21,22^. Additonally, epilepsy (EP), is characterized by clinical syndromes arising from abnormally synchronized neuronal discharges triggered by diverse factors. The etiology of EP encompasses genetic predispositions, structural damage, or functional irregularities within the central nervous system, including traumatic brain injuries, cerebrovascular diseases, brain tumors, and central nervous system infections. Research has indicated that this condition correlates with elements such as age and metabolic processes^23^.

Mendelian randomization (MR) is an analytical method, recently proposed, for assessing the causal relationship between exposure and outcome factors. It utilizes genetic instrumental variables, such as single nucleotide polymorphisms (SNPs), which are randomly assigned and not subject to any causality. This method controls for confounders while maintaining the constancy of the genes and their independence from disease progression. MR studies avoid the effects of reverse causality and can provide a scientific evaluation of the genetics of the relationship between exposure and outcome^24,25^. This study explored the genetic association between BMI and various neurological diseases, aiming to provide some ideas and evidence for disease prevention and treatment.

## Materials and methods

### Study design

**Figure 1:**
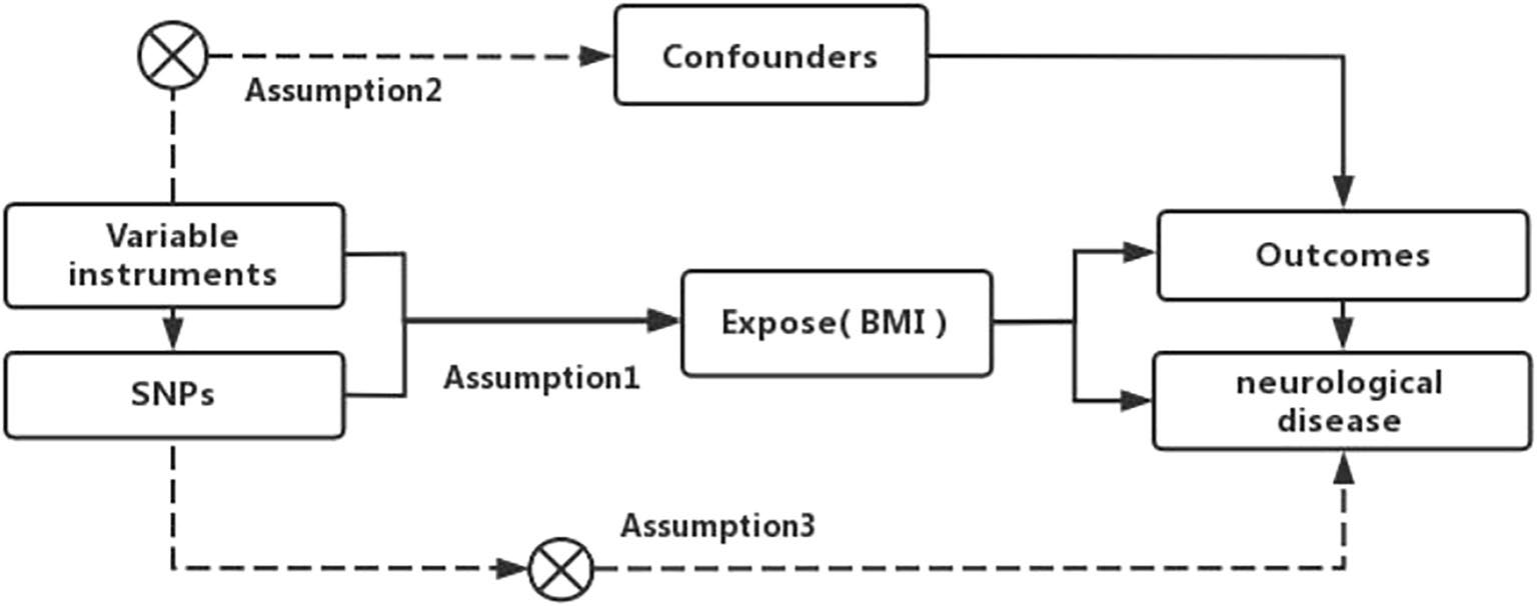
Diagram of the design of the two-sample Mendelian randomization study.

In this investigation, single nucleotide polymorphisms (SNPs) correlated with exposure and outcome factors within the Genome-Wide Association Study (GWAS) dataset were employed as instrumental variables to probe the genetic causality linking them. The study adhered to the tripartite hypothetical prerequisites for MR studies: (1) All instrumental variables exhibited a robust correlation with exposure factors, with underperforming instrumental variables omitted (*P* < 5 × 10^−8^, F-statistics > 10); (2) Instrumental variables bore no association with potential confounders, a premise corroborated by an exhaustive review of the literature and pertinent statistical methodologies; (3) All instrumental variables were stipulated to impact the outcome exclusively via BMI levels, precluding alternative pathways^26,27^. The data selected for this study was sourced from open-access public databases. An illustration of the study design is depicted in Fig. 1.

### Data sources

Data on BMI indicators were sourced from the IEU database, which contains genome-wide association summary data^28,29^ (available at https://gwas.mrcieu.ac.uk/datasets/ieu-b-4816/). The cohort comprised 999,998 male and female participants of European descent, with measurements for 7,191,606 SNPs. Ethical approval and informed consent were previously obtained in the original study, as documented in the relevant literature. The initial ethical guidelines adhered to the Declaration of Helsinki. All indicators about age, gender mismatch, non-European ancestry, and duplicate samples underwent stringent quality control. Subsequently, the identified SNPs were utilized for further analysis following covariate harmonization.

Summary genome-wide association data^30–35^ for multiple neurologic diseases were obtained from the IEU database (https://gwas.mrcieu.ac.uk/datasets/ieu-b-7/, https://gwas.mrcieu.ac.uk/datasets/ieu-b-2/, https://gwas.mrcieu.ac.uk/datasets/ieu-b-18, https://gwas.mrcieu.ac.uk/datasets/ebi-a-GCST90013429/, https://gwas.mrcieu.ac.uk/datasets/ebi-a-GCST0058), which included 33,674 PD cases, and 449,056 controls, 21,982 AD cases and 41,944 controls, 47,429 MS cases and 68,374 controls, 22,040 amyotrophic lateral sclerosis cases and 62,644 controls, 34,217 IS cases and 40,611 controls,15,212 EP cases and 29,677 controls. The participants with PD, AD, MS, and IS were of European origin, and those with amyotrophic lateral sclerosis and EP were a mixed multiracial and multiregional population. We have attributed and controlled the quality of all disease data above and obtained relevant genetic variations (nSNPs; PD = 12,858,066, AD = 10,528,610, MS = 6,304,359, ALS = 10,181,076, IS = 7,537,579, EP = 4,880,492). On this basis, SNPs related to BMI indicators were further extracted. All participants, sample size, genetic variation, and quality control information can be searched and obtained through the IEU database website.

### Selection of instrumental variables

By identifying SNPs that are significantly associated with the BMI index (p < 5 × 10^−8^), and subsequently conducting cluster analysis based on these SNPs (r^2^ < 0.001, cluster distance = 10,000 kb), we excluded SNPs with similar effects. Additionally, SNPs that were directly causally linked to the outcomes (PD, AD, MS, ALS, IS, EP; *P* < 5 × 10^−8^) were also excluded. By reviewing the literature, further exclusion of SNPs related to confounding factors was conducted, such as risk factors associated with PD: age, gender, coffee consumption, pesticides, smoking, alcohol consumption, uric acid levels, stroke, cardiovascular diseases, encephalitis, head injuries, and thyroid diseases^11,36–44^. Risk factors associated with AD: gender, age, smoking, diet, traumatic brain injury, cardiovascular diseases, diabetes, stroke, and hearing impairment^45–51^. Risk factors associated with MS: age, gender, smoking, birth season, EBV infection, and VitD^52–55^. Risk factors associated with ALS: gender, age, smoking, physical activity, alcohol consumption, environmental factors, viral exposure, and cyanide toxicity^56–61^. Risk factors associated with IS: gender, age, smoking, alcohol consumption, income, diabetes, and heart disease^62–65^. Risk factors associated with EP: brain infections, metabolic disorders, immunity, head trauma, stroke, brain tumors, hypoxia, and drug or alcohol poisoning^66–74^. To mitigate the impact of weak instrumental variables on the study’s outcomes, we selected only those instrumental variables with a statistic F value greater than 10. Furthermore, to ensure that SNPs influenced outcome factors exclusively through exposure factors, we omitted SNPs of moderate allelic frequency. In instances where particular SNPs were unavailable, we utilized an online platform to search for and obtain information on suitable proxy SNPs (https://ldlink.nci.nih.gov/).

### MR analysis

The association between BMI and PD, AD, MS, ALS, IS, and EP, was investigated through a genetic study. This study employed a two-sample MR analysis, utilizing the “TwoSampleMR” package in R language software, version 4.2.1. The primary analytical approach was the Inverse Variance Weighting (IVW) method, complemented by additional techniques such as MR Egger, Weighted Median (WM), Simple Mode, and Weighted Mode. IVW is a technique that aggregates multiple random variables to minimize the variance of their sum. It assigns weights to each variable inversely proportional to its variance, facilitating the combination of results from independent studies using the Wald ratio method. The Wald ratio method calculates exposure-outcome effect values for each SNP, providing a robust causality estimate between exposure and outcome, and is regarded as the principal analytical method^75,76^. The Egger regression method facilitates multivariate assessment of outcomes through its intercept. The MR-PRESSO (MR Pleiotropy Residual Sum and Outlier) method calculates the median of a distribution function derived from ranking SNP effect sizes based on their weights. This method is primarily used when at least 50% of the data originates from valid instrumental variables, resulting in more robust estimates. Compared to IVW, both simple and weighted modal assessments are less efficacious, rendering them supplementary and complementary analyses.

### Sensitivity analysis

We performed sensitivity analysis on the results of the causal relationship between BMI indicators and PD, AD, MS, ALS, IS, and EP, which mainly included horizontal pleiotropy, heterogeneity test, and Leave-one-out method^77–79^. The MR-Egger method was used to test whether the results had horizontal pleiotropy, and when the regression intercept was not zero and *P* > 0.05, it indicated that there was no sign of horizontal pleiotropy. The Cochran Q test was used to test for the presence of heterogeneity among individual SNPs by assessing the *P*-value, and when *P* > 0.05, it indicated the absence of heterogeneity, in which case the fixed-effects IVW method would be used, otherwise the random-effects IVW method would be used. The leave-one-out method was used to assess whether the combined effect of the remaining SNPs after the removal of one SNP was consistent with the main effect; if it was consistent, it indicated that the individual SNPs removed did not have an excessive effect on the MR analysis, then the result was considered to be reliable, and vice versa.

### Ethical approval and consent to participate

This study used only publicly available GWAS summary statistics and therefore did not require ethical approval. The relevant GWAS researchers have obtained their respective ethical approvals.

## Results

### Association estimation of SNPs for genome-wide significance of BMI and multiple neurological diseases

In accordance with the instrumental variable screening criteria employed in this study, the 7,191,606 SNPs associated with the BMI index and the 12,858,066 associated with PD, the 10,528,610 associated with AD, the 6,304,359 associated with MS, the 10,181,076 associated with ALS, the 7,537,579 associated with IS, and the EP related 4,880,492 SNPs were analyzed separately for coordination. A total of 42 SNPs associated with PD, 42 SNPs associated with AD, 39 SNPs associated with MS, 42 SNPs associated with ALS, 42 SNPs associated with IS, and 31 SNPs associated with EP, all exhibiting genome-wide significance for BMI indicators.

### Results of MR analysis

**Figure 2:**
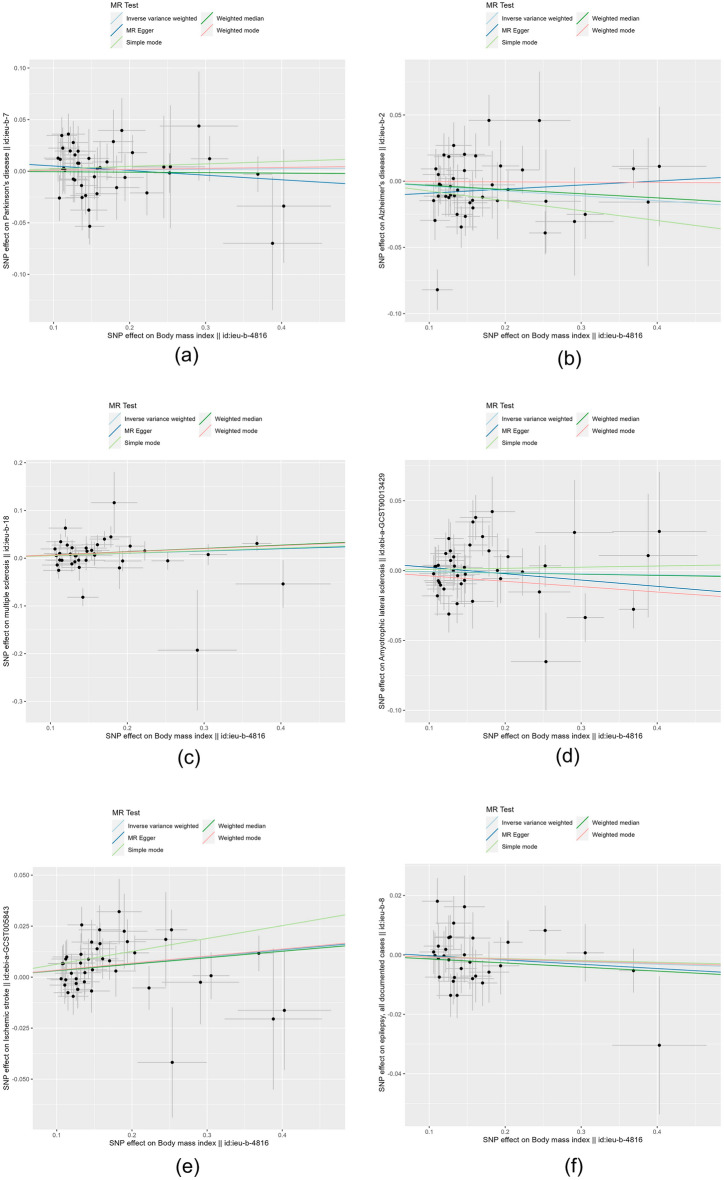
Scatter plots of the causal effect of BMI metrics with multiple neurological disorders ((**a**) PD, (**b**) AD, (**c**) MS, (**d**) ALS, (**e**) IS, (**f**) EP). The horizontal coordinate on the plot is the effect of SNP on BMI indicators (black dots are beta values, horizontal crosses are standard errors, and the vertical coordinate is the effect of SNP on diseases (black dots are beta values, vertical crosses are standard errors), and the slopes of the straight lines represent the causal effect of each method, respectively. Regression lines for inverse variance weighted (IVW), weighted median, MR-Egger, simple, and weighted models are shown.

**Figure 3:**
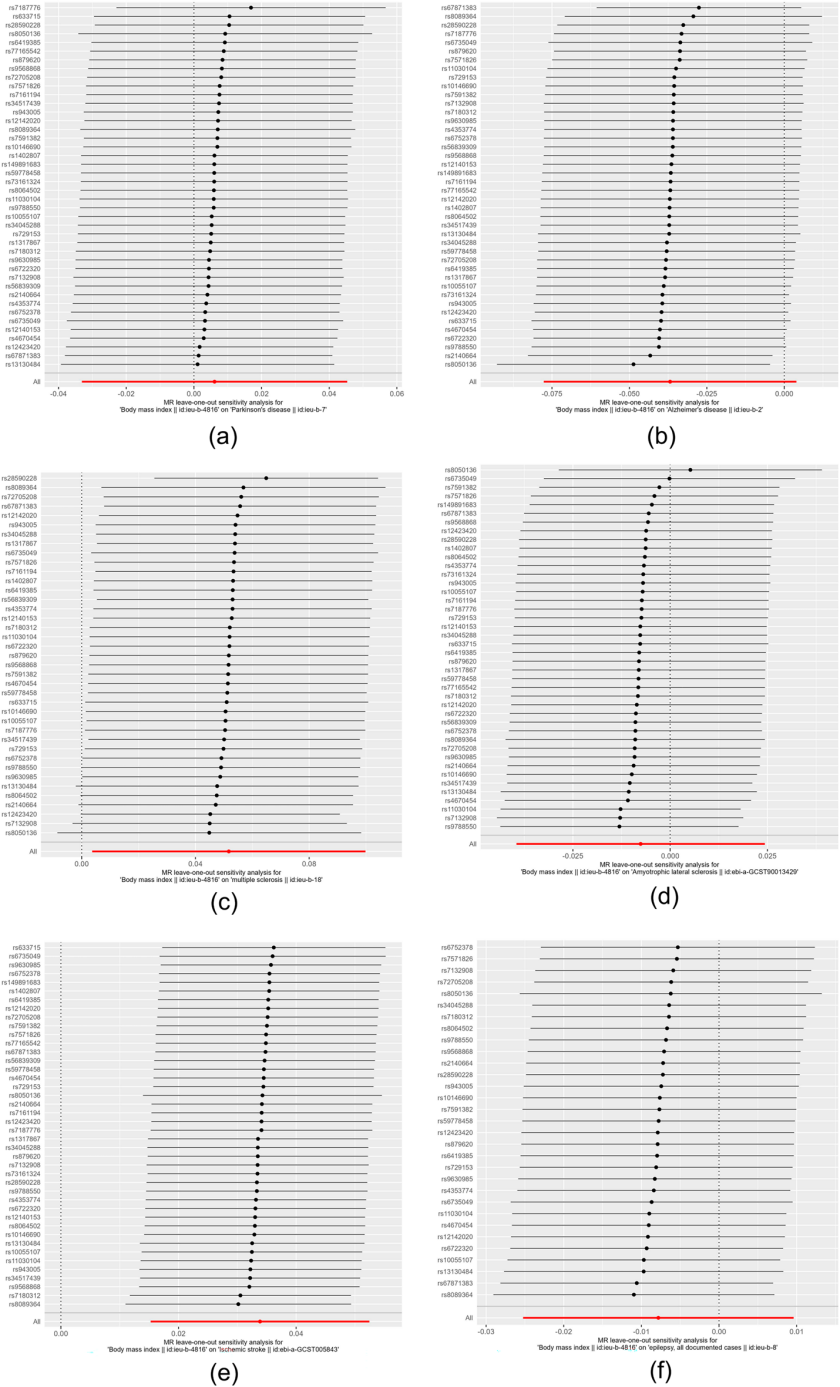
Forest plots of causal effects of BMI metrics with multiple neurological diseases ((**a**) PD, (**b**) AD, (**c**) MS, (**d**) ALS, (**e**) IS, (**f**) EP). Red and black dots and lines represented causal estimates of the risk of multiple neurological disorders by BMI metrics.

The results of IVW analysis suggested that there was no clear genetic causality between BMI index and PD (β = − 0.044 95% Cl − 0.175–0.087; *P*-value = 0.511), AD (β = − 0.037; 95% Cl − 0.078–0.004; *P*-value = 0.076 ), ALS (β = − 0.008; 95% Cl − 0.039–0.023; *P*-value = 0.641), epilepsy (β = − 0.008 95% Cl − 0.026–0.010; *P*-value = 0.380). Nevertheless, there was a positive genetic causality between BMI index and MS (β = 0.052; 95% Cl 0.003–0.101; *P*-value = 0.035), IS (β = 0.034; 95% Cl 0.016–0.052; *P*-value = 0.000) and BMI was positively associated with the risk of developing both. The results of MR Egger's analysis suggested that there was no causal relationship between BMI and PD, AD, MS, ALS, IS, and EP (*P* > 0.05). The results of the weighted median method indicated that there was a causal relationship between BMI and MS, IS (*P* < 0.05). Simple model results implied a relationship between BMI and IS (*P* < 0.05). The results of the weighted model revealed that no genetic causality existed between BMI and PD, AD, MS, ALS, IS, and EP (*P* > 0.05). The results are shown in Table 1. Scatter plots were used to visualize the results, in which the regression lines for IVW, weighted median, MR-Egger, simple mode, and weighted mode are shown in Fig. 2. The forest plot demonstrated that among the neurological diseases studied, only the red lines of MS and IS indicators were located on the “0” side, suggesting that the BMI indicators MS and IS had a significant effect (Fig. 3).

**Table 1:** Results of MR analysis of BMI and neurological diseases.

Outcome	Expose	MR Egger		Weighted median		Inverse variance weighted		Simple mode		Weighted mode	
		Β (95% Cl)	*P*	Β (95% Cl)	*P*	Β (95% Cl)	*P*	Β (95% Cl)	*P*	Β (95% Cl)	*P*
PD	BMI	− 0.045 (− 0.150, 0.062)	0.411	− 0.005 (− 0.066, 0.056)	0.879	0.006 (− 0.033, 0.045)	0.511	0.024 (− 0.078, 0.126)	0.652	0.009 (− 0.058, 0.076)	0.790
AD	BMI	0.031 (− 0.081, 0.143)	0.590	− 0.032 (− 0.079, 0.015)	0.196	− 0.037 (− 0.078, 0.004)	0.076	− 0.074 (− 0.174, 0.026)	0.155	− 0.002 (− 0.071, 0.071)	0.950
MS	BMI	0.048 (− 0.085, 0.181)	0.491	0.068 (0.011, 0.125)	0.019	0.052 (0.003, 0.101)	0.035	0.053 (− 0.043, 0.149)	0.286	0.066 (0.001, 0.130)	0.052
ALS	BMI	− 0.045 (− 0.133, 0.043)	0.318	− 0.009 (− 0.054, 0.036)	0.706	− 0.008 (− 0.039, 0.023)	0.641	0.008 (− 0.088, 0.104)	0.870	− 0.038 (− 0.107, 0.031)	0.284
IS	BMI	0.033 (− 0.018, 0.084)	0.212	0.032 (0.001, 0.063)	0.043	0.034 (0.016, 0.052)	0.000	0.063 (0.006, 0.120)	0.033	0.034 (− 0.005, 0.073)	0.089
EP	BMI	− 0.014 (− 0.061, 0.033)	0.552	− 0.014 (− 0.039, 0.011)	0.308	− 0.008 (− 0.026, 0.010)	0.380	− 0.006 (− 0.055, 0.043)	0.806	− 0.007 (− 0.042, 0.028)	0.697

### Sensitivity analysis of MR results

**Figure 4:**
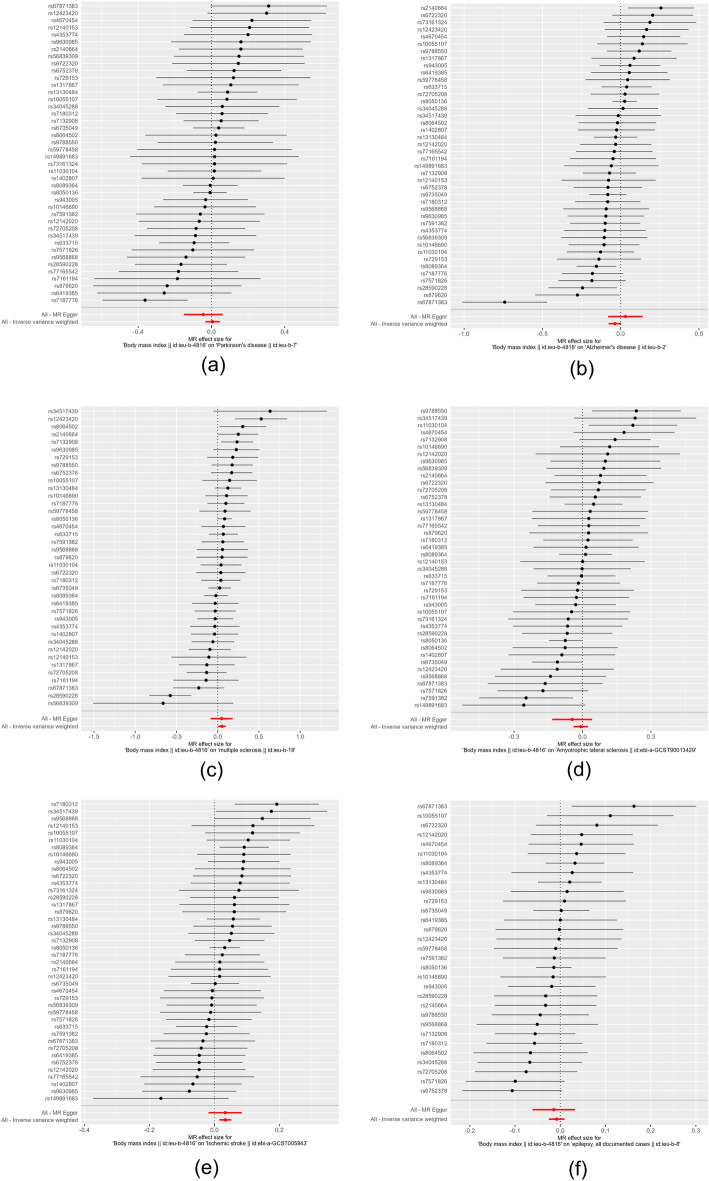
Funnel plot of causal effects of BMI metrics with multiple neurological diseases ((**a**) PD, (**b**) AD, (**c**) MS, (**d**) ALS, (**e**) IS, (**f**) EP), the blue vertical line is the causality estimation for all SNPs, and whether all black dots are symmetrically distributed on both sides of the line is used as a measure of the reliability of the MR results.

The results of Cochran's Q test in MR-IVW indicated that there was no significant heterogeneity between the results of MR analysis of BMI index and various neurological diseases (*P* > 0.05). The findings from the MR Egger’s test were in line with those from the Cochran's Q test. The funnel plots (Fig. 4) showed that the SNPs for MS and IS were relatively evenly distributed on both sides of the straight line in multiple neurological diseases. Additionally, the Egger intercept test indicated that no significant pleiotropic effects were detected in any of the neurological diseases examined (*P* > 0.05). MR presso test further corroborated this finding (Table 2).

**Table 2:** Results of sensitivity analyses of BMI and MR for multiple neurological diseases.

Outcome	Expose	Heterogeneity test		Pleiotropy test	Mr presso test
		Cochran’s Q test (*P* value)	Rucker’s Q test (*P* value)	Egger intercept (*P* value)	Mr presso test
		Inverse variance weighted	MR Egger	MR Egger	*P* value
PD	BMI	0.633	0.636	0.315	0.654
AD	BMI	0.002	0.003	0.207	0.002
MS	BMI	0.004	0.003	0.595	0.003
ALS	BMI	0.153	0.150	0.371	0.142
IS	BMI	0.560	0.515	0.977	0.566
EP	BMI	0.586	0.539	0.768	0.628

**Figure 5:**
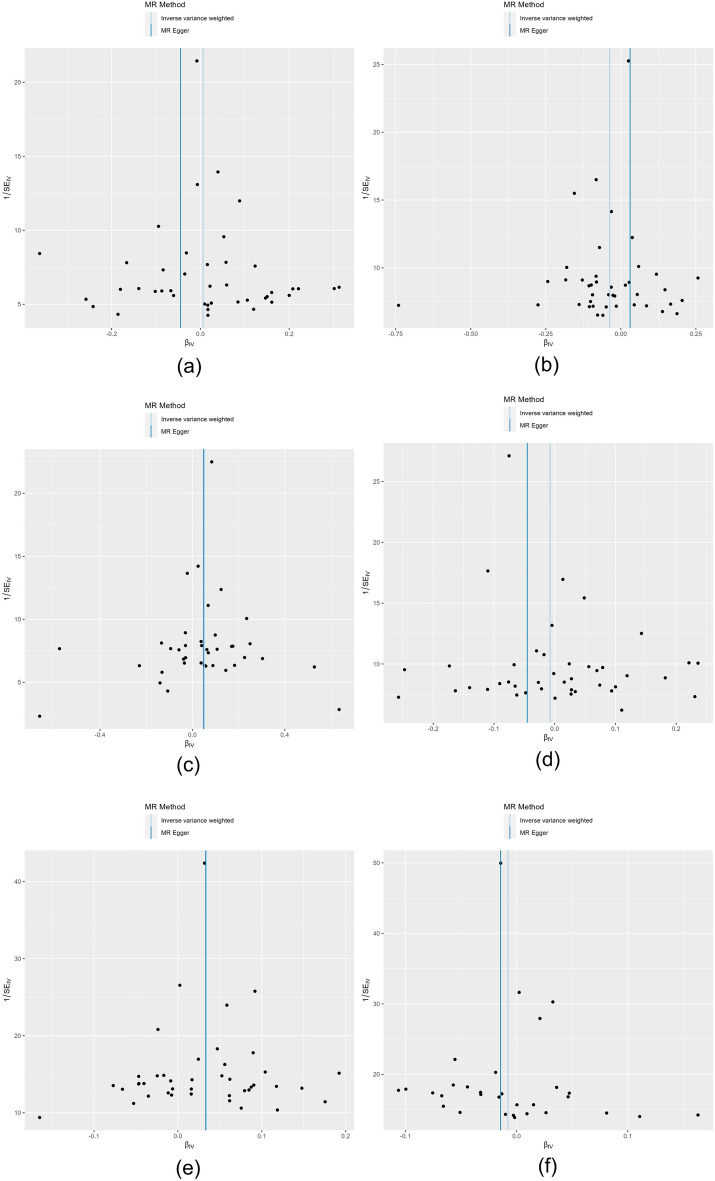
Leave-one-out method to exclude the effect of individual SNPs on the overall results ((**a**) PD, (**b**) AD, (**c**) MS, (**d**) ALS, (**e**) IS, (**f**) EP). The black dots in the lines in the figure indicate the results of the IVW method after excluding a particular SNP, and the red dots indicate the estimation of the results of the IVW method for all SNPs.

By visualizing and expressing the results of the IVW method through the Leave-one-out method and eliminating individual SNPs sequentially, it becomes evident that the distribution of the remaining SNPs associated with the investigated neurological disorders remains proximal to the red-marked position on the graph. The aggregate findings remain largely unperturbed, thereby corroborating the stability and reliability of the IVW analytical results, as depicted in Fig. 5.

## Discussion

In this study, we examined the genetic associations between BMI indicators and various neurological diseases for the first time. All data were obtained from genome-wide association data. Following rigorous quality control, we identified SNPs associated with BMI and extracted SNPs linked to various neurological disorders (PD, AD, MS, ALS, IS, EP). Based on these findings, we further analyzed and categorized SNPs related to both BMI and the aforementioned diseases, yielding millions of quantitative genetic variants. By conducting MR and sensitivity analyses, the results suggested that there was a genetically causal relationship between BMI and MS (*P* value = 0.035) and IS (*P* value = 0.000) and that BMI was positively associated with the risk of developing both. There was no clear genetic causal relationship between BMI and PD (*P* value = 0.511), AD (*P* value = 0.076), ALS (*P* value = 0.641), and epilepsy (*P* value = 0.380). Sensitivity analysis suggested no heterogeneity and horizontal pleiotropy in the results of MR analysis between BMI index and the neurological diseases studied (*P* > 0.05).

In this study, we identified a genetic causal relationship between BMI indicators and MS as well as IS, with a positive correlation to the risk of morbidity. This finding aligns with previous research. MR studies in recent years have indicated that an increased BMI elevates the risk of MS, confirming obesity's causal role in the etiology of MS^80–82^. The main pathological mechanisms are unclear, and studies have shown that this may be related to the increased systemic organismal inflammatory response caused by obesity, which has a promoting effect on the development of autoimmune responses^83–85^. Morales et al.^86^ demonstrated significant improvements in inflammation, demyelination, and axonal damage significantly in mice, suggesting that intermittent fasting (IF) has neuroprotective and anti-inflammatory effects, which may be related to the modulation of intestinal bacterial abundance degree and metabolite changes. It has been discovered that BMI can influence MS by affecting specific hormone levels in the body, including lipocalin and leptin. Notably, leptin, as an essential immune system regulator, can effectively prevent the migration of immune cells and reactions to the central nervous system via receptor blockade, improving the progression of experimental autoimmune encephalomyelitis (EAE)^87,88^. Additionally, it has been associated with macrophage phagocytosis and the regulation of pro-inflammatory factor release^89,90^. Further investigation into additional mechanisms is necessary. Our findings also implied a causal relationship between BMI indicators and IS, consistent with most previous research. A retrospective study from 2015 revealed that obesity could increase the risk of IS, possibly due to the impact on blood pressure, blood glucose, and other relevant indicators^91^. Recent research has shown that a high BMI in adolescents correlates with an elevated risk of youthful stroke^92^. This may result from visceral fat deposition caused by a high BMI, triggering inflammatory responses and oxidative stress, impairing vascular endothelial function, and thus inducing thrombosis^93,94^. Moreover, fat deposition can affect liver and muscle metabolism in humans, leading to insulin resistance and consequent elevations in blood glucose levels, damaging the vascular endothelium^95^. A small number of studies suggest that obese patients might improve their prognosis after IS by enhancing the early-stage anti-inflammatory response^96^. Rodríguez and Liu^97,98^ propose that a high BMI might increase the survival rate of IS patients, potentially due to better metabolic reserves. More clinical studies and experimental validation are required in the future to clarify its mechanism.

The current study failed to establish a link between BMI and PD. Earlier investigations in this domain have yielded conflicting results. Wang et al.^99^ and others posited that elevated BMI does not elevate the risk of PD, while Yoon et al.^100^ found that women with lower BMI exhibited higher mortality rates compared to men with PD. Conversely, certain studies have presented opposing conclusions. A MR study previously indicated a causal relationship between reduced BMI and PD^101^. A meta-analysis suggested that being overweight or obese might act as a risk factor for PD, potentially triggering the onset of the condition^102^. Wills et al.^103^ concluded that no correlation exists between BMI and PD mortality. The precise mechanism underlying these observations remains elusive. No causal relationship was identified between BMI and AD in this study. Research in this area is currently inconclusive. Findings suggest that higher adult BMI or obesity is not linked to the progression of AD^51,104^. Nordestgaard et al.^105^ suggested that low BMI is not a risk factor for AD and is not associated with an increased risk of developing AD. However, contradictory findings propose that higher BMI correlates with a decreased risk of AD among individuals sharing the same genetic susceptibility^106^. It has also been postulated that both high and low BMI could augment the risk of AD development^105^. This may be attributed to obesity-induced inflammatory responses that further compromise endothelial function and the blood–brain barrier, consequently affecting cognition^107,108^. Moreover, research has uncovered that high BMI in premenopausal women can escalate the risk of AD, which might be associated with hormonal levels, inflammation, and other metabolic factors. Another study confirmed that after menopause, high BMI can reduce the risk of AD in women. The researchers suggested that this may be due to changes in body composition and metabolism in postmenopausal women^109,110^. Therefore, further research is necessary to establish a clear association and mechanism between the two variables. The current study found no correlation between BMI and ALS. There has been inconsistency regarding the results of studies in this area. A MR study demonstrated that genetically increasing or decreasing BMI had no causal effect on the risk of developing ALS^111^. Conversely, a prospective study indicated that higher BMI and weight gain were associated with a lower risk of ALS after 50 years. O'Reilly discovered that a lower pre-morbid BMI increased the risk of ALS^112^. Military personnel and athletes, particularly football players, appear to have a higher risk of ALS. This may be attributed to muscle damage caused by strenuous exercise, free radical production, or other unknown factors. However, this does not imply that exercise is detrimental^113,114^. Early epidemiology has identified a decrease in body mass index early in the onset of ALS^115,116^, but this merely reflects the symptoms and prognosis of the disease after its onset, and the effect of pre-morbid BMI on the disease requires further investigation. This study did not find a causal link between BMI and EP. Experts hold varying views on this matter. Some scholars have argued that there was no significant difference in exercise participation rates between epileptic and non-epileptic patients^117,118^, refuting the impact of obesity on EP development. A recent prospective study concluded an association between obesity and idiopathic generalized EP and a family history of EP, but not drug-resistant EP^119^. A MR analysis investigated the relationship between obesity, institutional distribution, and EP subtypes and found that obesity, a risk factor for EP, can lead to an increased risk of spasmodic seizures in adolescents, while a high BMI raised the risk of cataplectic seizures in children^120^. Early studies identified the possibility of co-morbidity between EP and obesity, suggesting a risk of obesity-induced EP^121–123^. Indeed, the early discovery of the serotonin 5ht2c receptor is not only involved in organismal obesity^124^ but has been recognized as a potential target for antiepileptic drug action^125^. Therefore, we hypothesized that the reason for the association between high BMI and EP may be related to neurological dysfunction caused by mutations in some specific genes. However, our study did not show positive results, which may be due to factors such as sample size and ethnicity of origin. The exact mechanism needs to be further clarified and studied.

In the current investigation, we utilized the GWAS database to offer a precise assessment of the genetic linkage between exposure and outcomes. This was achieved by identifying instrumental SNPs that exhibit robust associations with both exposure and outcome factors. Our approach circumvented the influences of confounding variables and the reverse causation inherent in transcriptional processes. Additionally, we employed a database derived from an open and dependable source^24,25^. Therefore, the use of the MR method is feasible and has good value in providing a more reliable genetic assessment of the association between BMI and various neurological disorders.

## Limitations

There are several limitations to this study. First, most of the data involved in this study were patients of European ancestry, which biases the results somewhat and needs to be validated by including more ancestries and ethnicities in the future. Secondly, numerous risk factors are associated with neurological disorders. Despite our efforts to remove all factors that can be accessed, we still cannot fully cover them, which may have a certain impact on the study. Third, BMI is a simple index that measures the relationship between weight and height, but it does not distinguish the proportion of fat and muscle in body weight, nor does it reflect important health factors such as body fat distribution or insulin sensitivity. In future studies, other indicators such as waist circumference, waist-to-hip ratio, body fat percentage, and bioelectrical impedance analysis should be included to prevent bias in results from a single indicator.

## Conclusion

In summary, this study investigated the genetic associations between BMI and various neurological diseases using MR analysis. The findings indicated that there was no causal relationship between BMI and PD, AD, ALS, and EP. The study found that there was a genetic association between BMI and MS as well as IS, and a higher BMI increased the risk of MS and IS. The findings underscore the significant role of obesity as a risk factor for certain neurological disorders, influencing their onset and progression. These insights are anticipated to offer a foundational framework for the prevention, treatment, and prognosis of these conditions.

## Data Availability

The data for this study is sourced from public databases, and relevant data and materials are available from corresponding GWAS databases.
